# Using Probabilistic Cellular Automata to Model the Transmission of an Emerging Infectious Disease of Amphibians—A Preliminary Model

**DOI:** 10.3390/pathogens15080827

**Published:** 2026-08-06

**Authors:** Amanda Linda Jean Duffus, Joshua Paul Standridge, Patricia Lillian Bartlett, John Clay George

**Affiliations:** 1 Health of Herpetofaunal Communities Research Group, Department of STEM, School of Nursing, Health, and STEM, Gordon State College, Barnesville, GA 30204, USA; 2 Department of Information Technology, Gordon State College, Barnesville, GA 30204, USA; 3 Department of STEM, School of Nursing, Health, and STEM, Gordon State College, Barnesville, GA 30204, USA; jgeorge@gordonstate.edu

**Keywords:** ranaviruses, common frogs, Python, amphibians, declines, disease emergence

## Abstract

Emerging infectious diseases are major threats to amphibian biodiversity. Ranaviruses (Family *Iridoviridae*) are a group of globally emerging infections that infect poikilothermic vertebrates. In the United Kingdom, the emergence of this group of viruses has been followed by the decline in populations of common frogs (*Rana temporaria*) across the country, especially in the southeast of England. Simple susceptible-infected (SI) and combinatorial models have been developed to explain the spread of ranavirus in populations of common frogs; here, we use those models as a basis for developing a simple transmission model using cellular automata with the programming language Python.

## 1. Introduction

Emerging infectious diseases have the potential to devastate biodiversity by causing both local extirpations and extinction [1,2,3]. Amphibians have been particularly hard hit by emerging infectious diseases around the globe. One fungal pathogen, *Batrachochytrium dendrobatidis*, is thought to be responsible for the decline in over 500 species and the extinction of 90 others [4]. However, there are other pathogens that have also caused declines and local extirpations in amphibians and one of them are the ranaviruses [5,6,7]).

Ranaviruses, members of the family *Iridoviridae*, are large double-stranded DNA viruses [8] and are globally emerging pathogens of poikilothermic vertebrates [9]. They began to emerge in the United Kingdom in the late 1980s [10] and are prevalent in amphibians around the globe, affecting over 170 species [9], although this is likely to be an underestimate. The ranaviruses present in the UK predominantly affect and have been studied in common frog (*Rana temporaria*) adults that have two different disease syndromes, which are not necessarily mutually exclusive [10]. The ulcerative form is characterized by ulcerations that may involve the musculature below, and the hemorrhagic form is characterized by severe internal hemorrhages, most notably in the gastrointestinal and urogenital tracts ([10]; Duffus Personal Observation). It is interesting to note that, despite high mortality rates, adult-to-adult transmission alone is sufficient to maintain the infection in a population [11]. In the UK, tadpoles of common frogs are not known to be infected readily with ranaviruses in the wild [12], which is an unusual situation. In North America, it is more common to find ranavirus infections in tadpoles/larval stages of amphibians (e.g., [13,14]). The emergence of ranaviruses in UK common frog populations has been accompanied by population declines where the pathogen has become established [5]. The average population decline is a striking 81% over a ten-year period [5]. Duffus et al. [11] found that despite these declines, ranavirus infections can persist in populations of common frogs for long periods of time.

Cellular automata have long been used to model the spread of infectious diseases, especially in humans (e.g., [15]). They can also be applied to infectious diseases that occur in animals (e.g., foot and mouth disease; [16]). Models have been used to explore the transmission dynamics of *B. dendrobatidis* (e.g., [17]). Mathematical models have also been used to explore ranavirus dynamics in populations of amphibians, such as wood frogs (*Lithobates sylvaticus*) in North America [18]. However, the use of cellular automata to analyze *B. dendrobatidis* or ranavirus dynamics in populations has not been attempted to our knowledge.

To date, researchers have used both susceptible-infection (SI) and combinatorial models to describe the persistence of ranavirus infections in UK common frog populations. Duffus et al. [11] use SI models to examine the persistence of ranavirus infections in common frog populations under several different scenarios, whereas Adams et al. [19]) examine ranavirus transmission using a combinatorial model. However, both models are limited in that they do not account for the many probabilistic occurrences found in such a complex system. To expand on these models, we look to probabilistic cellular automata, which can be extremely valuable in modelling the spread of infectious diseases on both large and small scales [20,21]. Below, we redefine the combinatorial model originally described by Adams et al. [19] and expand on it using elements of cellular automata in a computer program developed using Python 3.12.4 for 64-bit Windows (AMD 64; [22]).

## 2. A Combinatorial Prototype

There is growing evidence that the different disease syndromes may be caused by different strains of *Ranavirus* present in the UK [23,24,25,26]. Ranavirosis in the UK can exist in multiple states of disease/infection in the same population ([10]; Duffus Personal Observation); therefore, we must consider these in our models. While this evidence is currently not conclusive, we will treat them separately out of an abundance of caution. This decision is supported by Duffus et al. [11], who were able to derive different transmission rates for each syndrome from the literature (see below for more details).

Therefore, we consider the combinatorial prototype described by Adams et al. [19] that allows us to incorporate the two separate disease syndromes to make our transmission model. There are five possible states in which an individual frog may exist:

- Susceptible (***S***): an otherwise healthy frog that is susceptible to infection from both syndromes of the virus;
- Ulcerative (***U***): a frog that is infected with only the ulcerative syndrome;
- Hemorrhagic (***H***): a frog that is infected with only the hemorrhagic syndrome;
- Combination (***C***): a frog that is infected with both the ulcerative and hemorrhagic syndromes;
- Dead (***D***): a frog that has died from its infection(s).

**Figure 1:**
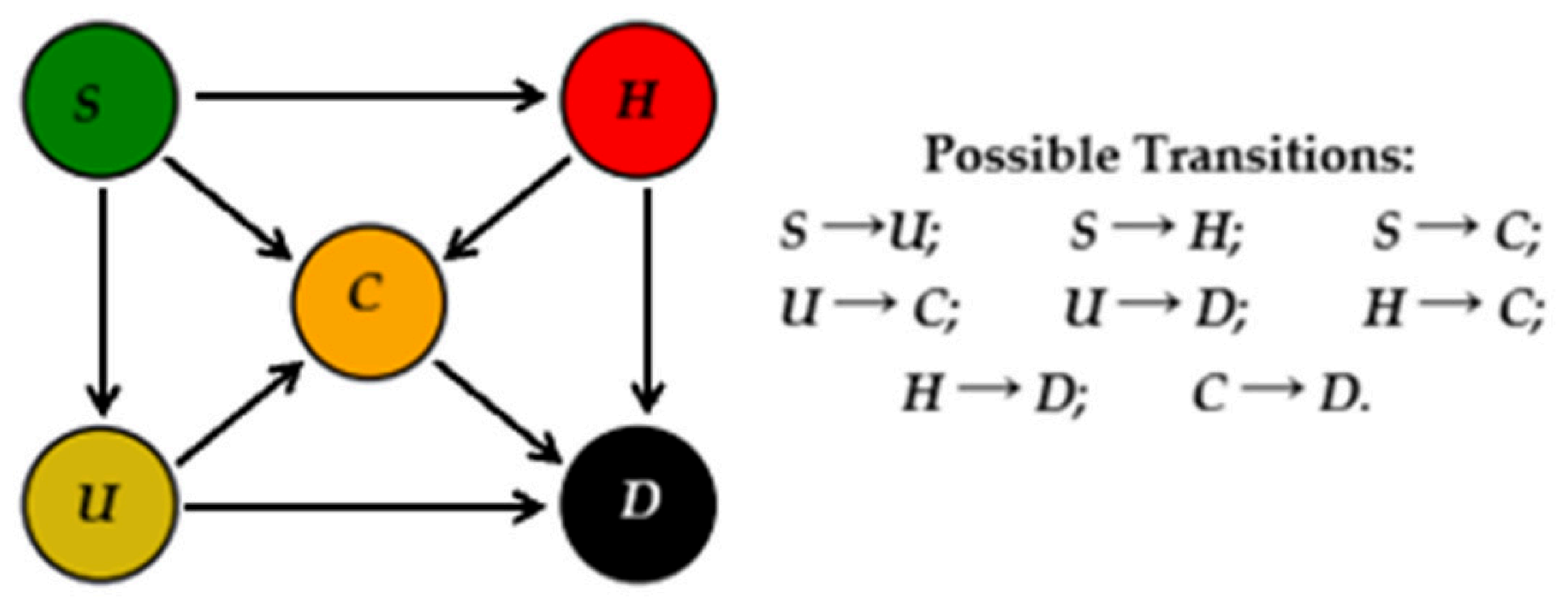
Digraph showing each possible transition based on infection/disease status or death (A→B denotes a transition from state ***A*** to state ***B***). We use green to indicate the Susceptible state, yellow to indicate the Ulcerative state, red for the Hemorrhagic state, orange for the Combined state, and black to indicate Death. This image is based on information from Adams et al. [19] and Duffus et al. [11] as well as the current model.

We allow frogs to transition from one state to another after each iteration. We outline the conversions an individual frog can experience in Figure 1. Notice that once a frog has contracted the infection, it may never return to ***S***, as this would imply a recovery, which is rare. Likewise, a frog may not transition from ***H*** to ***U*** or vice versa. Finally, we assume that a frog does not change its state within a given iteration (see Figure 1).

We can also use the digraph in Figure 1 to count the number of ways a frog might arrive at any particular state after *n* iterations. If we raise the adjacency matrix M of a graph to the *n*-th power, then the entries of the resulting matrix $M^{n}$ will be the total number of paths of length *n* from one vertex to another [27].

$$M=\left[\begin{array}{cc} \begin{array}{cccccc} & S & U & H & C & D\\ S & 1 & 1 & 1 & 1 & 0\\ U & 0 & 1 & 0 & 1 & 1\\ H & 0 & 0 & 1 & 1 & 1\\ C & 0 & 0 & 0 & 1 & 1\\ D & 0 & 0 & 0 & 0 & 1 \end{array} & \end{array}\right];\;M^{2}=\left[\begin{array}{cccccc} & S & U & H & C & D\\ S & 1 & 2 & 2 & 4 & 3\\ U & 0 & 1 & 0 & 2 & 3\\ H & 0 & 0 & 1 & 2 & 3\\ C & 0 & 0 & 0 & 1 & 2\\ D & 0 & 0 & 0 & 0 & 1 \end{array}\right]$$

We use this model to estimate the probability of a frog being in a specific state after a given amount of time. For example, the matrix $M^{2}$ shows that after two iterations, a frog starting in ***S*** has four ways to end up in ***C*** and eight ways to avoid ***C***, suggesting $\;P\left(S\to C\right)=\frac{4}{12}=\frac{1}{3}$. However, this is a naïve approach, because some of the paths represent scenarios that are more likely to happen than the others. In addition, the likelihood of each transition occurring is different for individuals depending on their current state and surroundings, both of which are subject to change over time. This means we cannot simply assign fixed weights to the edges of our digraph representing probabilities and use the same method. From this, it is clear that our model requires further investigation into the factors affecting the spread of the virus.

## 3. Hop ‘Til You Drop—A Probabilistic Cellular Automata-Based Model

Typically, common frog populations that have been affected by ranavirus emergence are found in isolated garden ponds in urban and suburban settings [5]. Building on the assumptions made in the earlier combinatorial prototype, we designed a program intended to emulate the spread of ranaviruses through a frog population. We arranged one hundred circles (each representing an individual frog) in a 10 by 10 grid and colored them according to their current infection state. We started our simulation with the entire population in state *S* and introduced both syndromes related to infection to one unlucky cell. Then, as our simulation ran, we used a pseudo-random number generator to determine whether the frogs/cells changed infection states. The probability that a frog made contact with an infection-carrying frog is based on both the demographics of the neighboring cells and the contact rate (ψ). We used ψ=1 for simplicity, and because other values of ψ could be simulated by appropriate reductions in the probability of transmission; e.g., for ψ=0.8, we would replace $\sigma_{U}$ by 0.65⋅0.80=0.52.

Furthermore, should any given frog encounter either disease syndrome, the probability of transition into a diseased state depends on the transmission rates of the syndrome $(\sigma_{U},\sigma_{H}$; Ulcerative and Hemorrhagic, respectively). Calculations made by Duffus et al. [11] suggest the following transmission rates: $\sigma_{U}=0.65$, and $\sigma_{H}=0.75$. We also assume that the individuals contact each of their neighbors once per iteration, and that the event of transmission of one syndrome is independent of the event of transmission of the other. However, these are extremely crude estimates, and further experimental work is necessary to determine more realistic estimates for these values.

**Figure 2:**
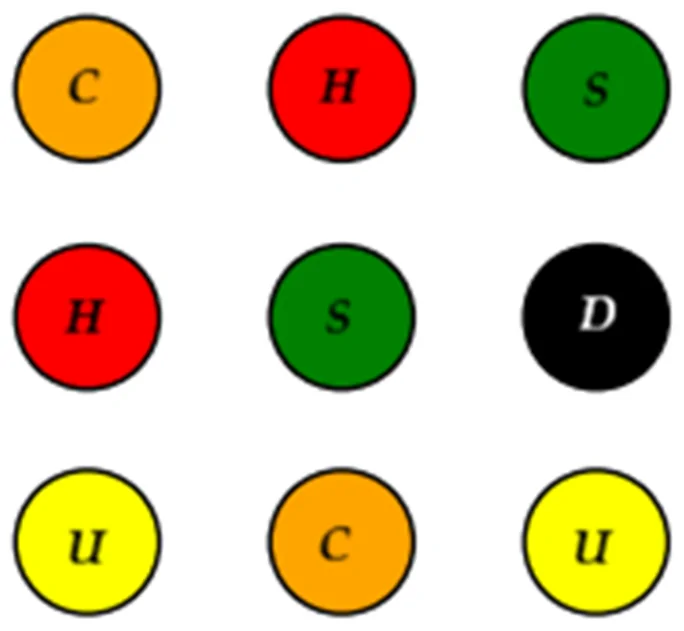
Illustration of a sample population showing how our model illustrates the state of each individual cell. (S in green cells = susceptible; U in yellow cells = Ulcerative; H in red cells = Hemorrhagic; C in orange cells = combination of H and U; D in the black cell indicates that the frog has died in a previous pass). The algorithm runs through each cell in turn to determine whether that cell’s state changes. When the green cell at the center of the figure is examined, we check (via pseudo-random number generator) to see if one or more of the neighboring cells spread one or both syndromes.

How our program calculated the transition probabilities of a given cell in a hypothetical scenario is shown in Figure 2. In the example, since four of the frogs surrounding the center cell carry the ulcerative form (*U* or *C*), the center cell has four opportunities to contract the ulcerative form. The two orange cells and the two yellow cells each have the opportunity to spread the ulcerative form and will do so with probability $\sigma_{U}=0.65$. Similarly, the four cells in either orange or red can spread the hemorrhagic form, giving four chances with probability $\sigma_{H}=0.75$. The dead cell, labeled *D*, cannot spread either syndrome. In this example, because the center cell has four neighbors (two ***U***, two ***C***) that can induce the ulcerative variety, the probability that at least one of them succeeds is given by $1-(1-0.65{)}^{4}=0.985$. With only three such neighbors, we would have $1-(1-0.65{)}^{3}=0.957$. Rather than compute these probabilities, we simply test each neighbor to see whether it passes on the ulcerative syndrome.

**Figure 3:**
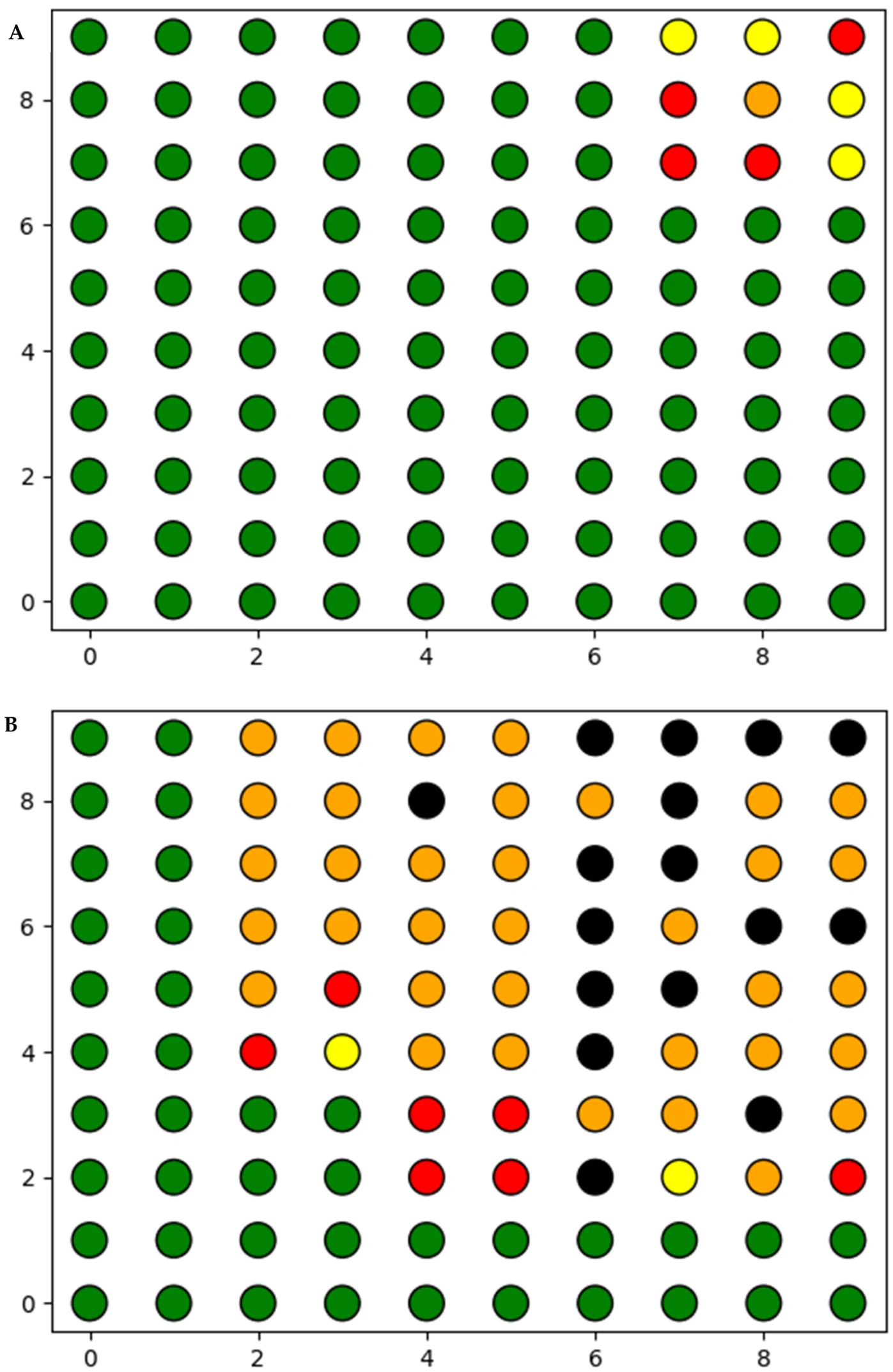

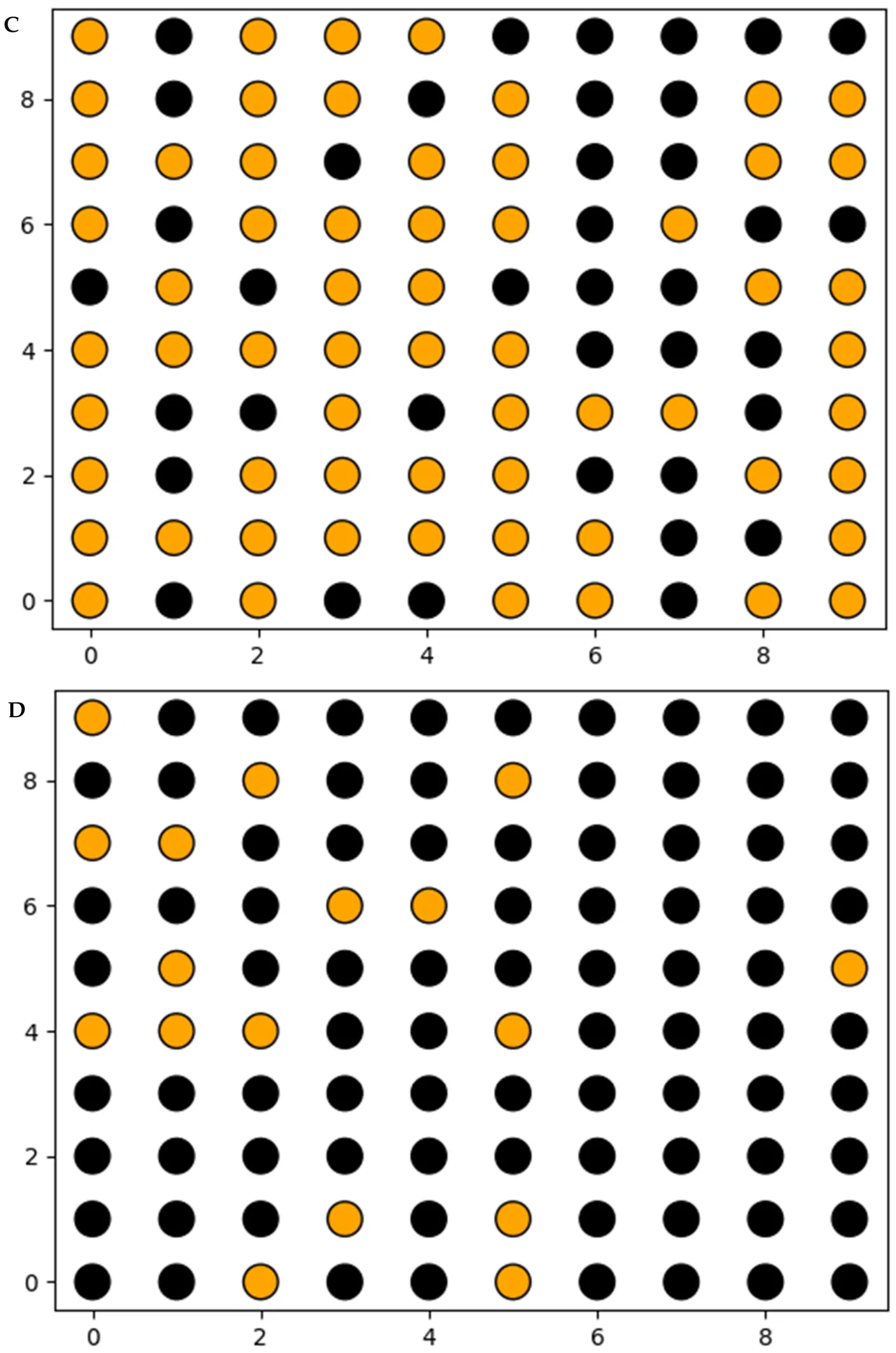
An example of the Hop ‘Til You Drop model. (**A**). After one iteration; (**B**). After six iterations; (**C**). After 11 iterations, there are no healthy frogs left; (**D**). After 36 iterations, there are only 17 survivors, all of which are infected by both syndromes. After 51 iterations, there are only 6 living frogs left, all affected with both syndromes. The green, yellow, red, and orange cells each represent a frog in a susceptible, ulcerative ($\sigma_{U}=0.65$), hemorrhagic ($\sigma_{H}=0.75$), and combination state, respectively. Black cells indicate that the individual has died (probability of 0.15 each iteration). The disease-induced mortality of 0.15 has been added to account for the 81% decline over a 10-year period that was reported by Teacher et al. [5]. Here we also assume that the frog contacts each of its neighbors once per iteration.

A visual representation of what takes place during these simulations is found in Figure 3. Keep in mind that simulations may vary wildly, as random events that take place early in a given trial may drastically change the outcome. The Python code may be found in the Supplementary Materials.

**Figure 4:**
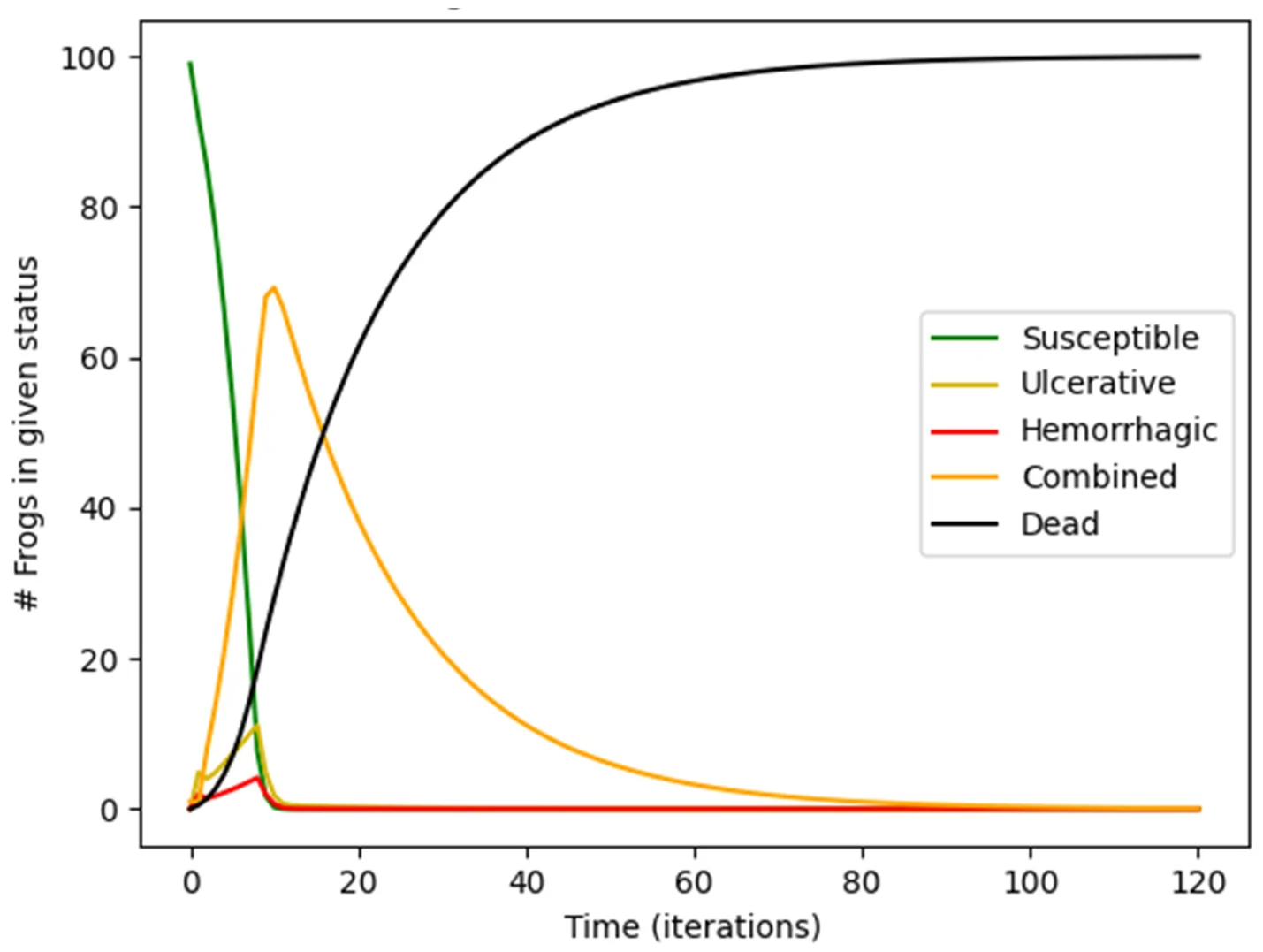
The average of 10,000 simulations of the Hop Til’ You Drop model over 120 iterations. We used $\omega =0.15,\;\sigma_{U}=0.65,\;\mathrm{and}\;\sigma_{H}=0.75$. Among the 10,000 runs, we found that there were no healthy (state ***S***) frogs left after 10 iterations on average, while there were no living frogs left after about 38 iterations on average among the 9626 ponds that had no survivors after 120 iterations.

The mean number of frogs in each state after each iteration, averaged over the 10,000 runs of the program, is plotted in Figure 4 below. The number of frogs with only the ulcerative syndrome is shown in the yellow curve, close to the bottom of the graph; the red curve shows the number with the hemorrhagic syndrome. Both curves are fairly low because a given frog does not remain infected with only one syndrome of the virus for long; consequently, the orange curve reflects the number of frogs with both syndromes, peaking at about 70 individuals.

As the simulation progresses, more frogs die off, and the black curve reflects this as it approaches the 100 mark asymptotically. Due to the skewness of the distribution, however, there were over 350 runs among the 10,000 in which some frogs still survived after 120 iterations.

**Figure 5:**
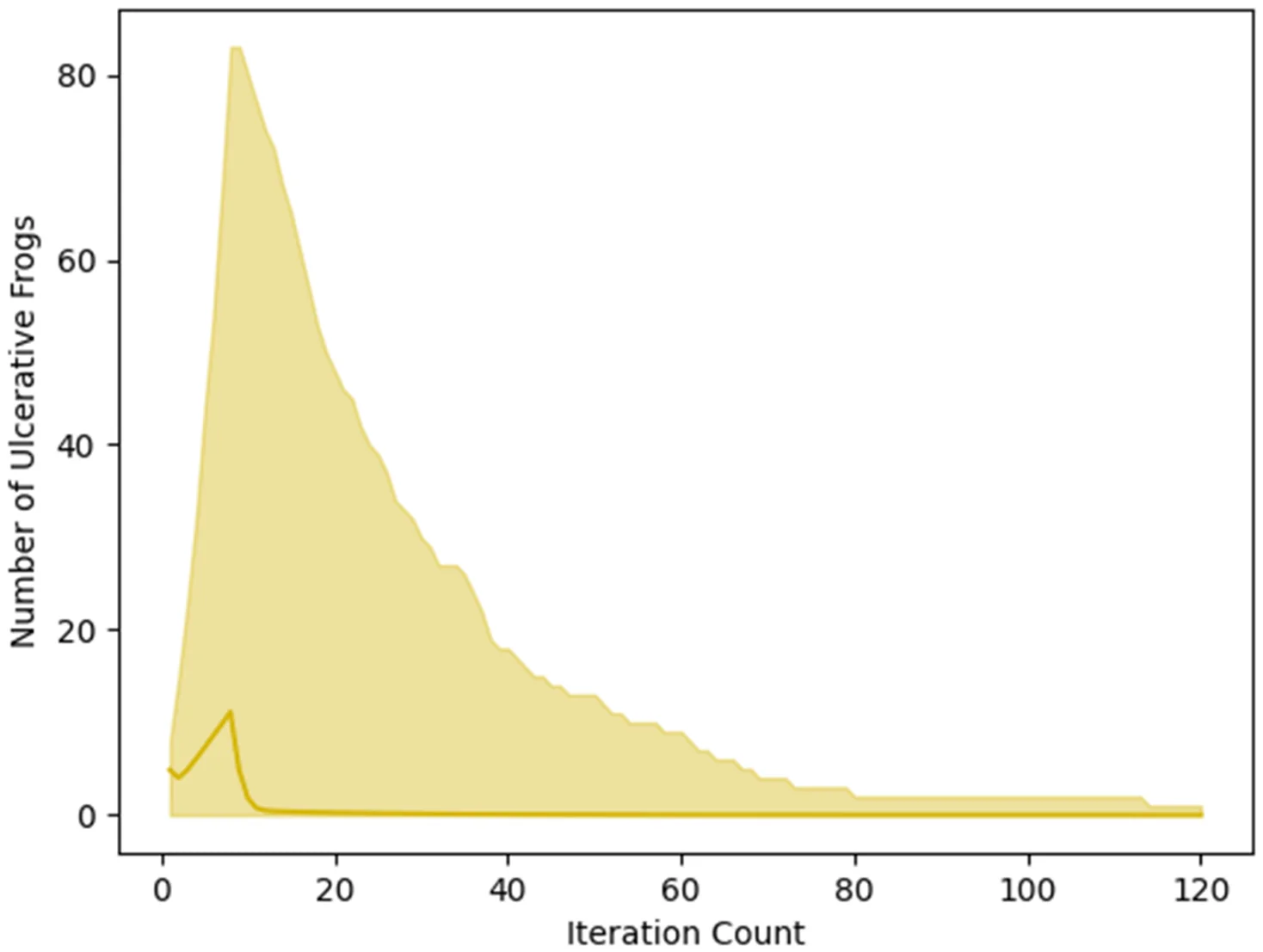
The dark yellow curve is the mean value of the number of frogs in state ***U*** over all 10,000 ponds for each iteration. The shaded region ranges from the minimum over all 10,000 ponds to the maximum. We note that there is no iteration count, from 0 to 120, for which no pond had zero frogs in state ***U***. This is due to the large variability of outcomes over this large number of ponds.

**Figure 6:**
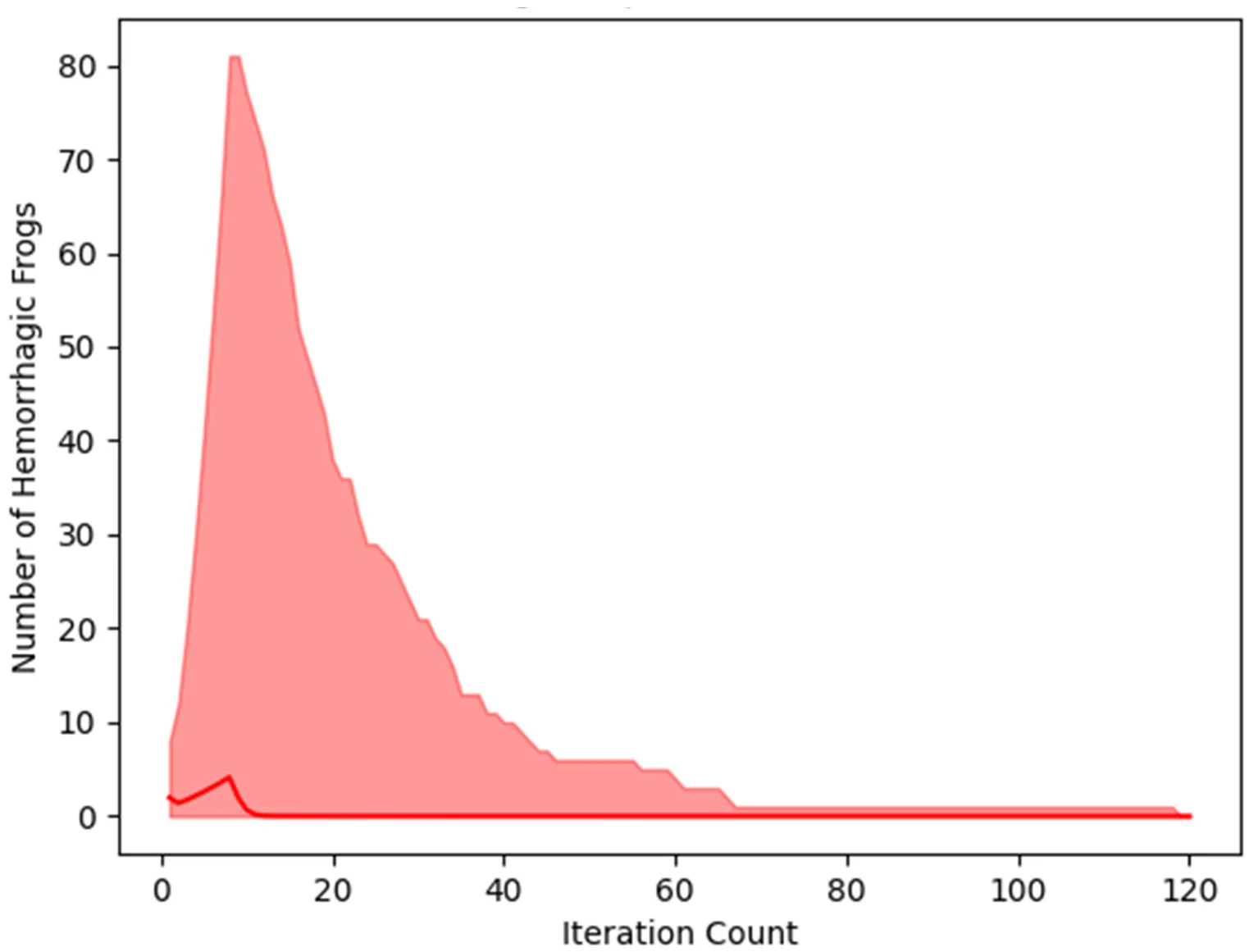
The dark red curve is the mean value of the number of frogs in state ***H*** over all 10,000 ponds for each iteration. The shaded region ranges from the minimum over all 10,000 ponds to the maximum. As before, we see that for each iteration count, there is at least one of the 10,000 ponds in which no frog is in state ***H***.

**Figure 7:**
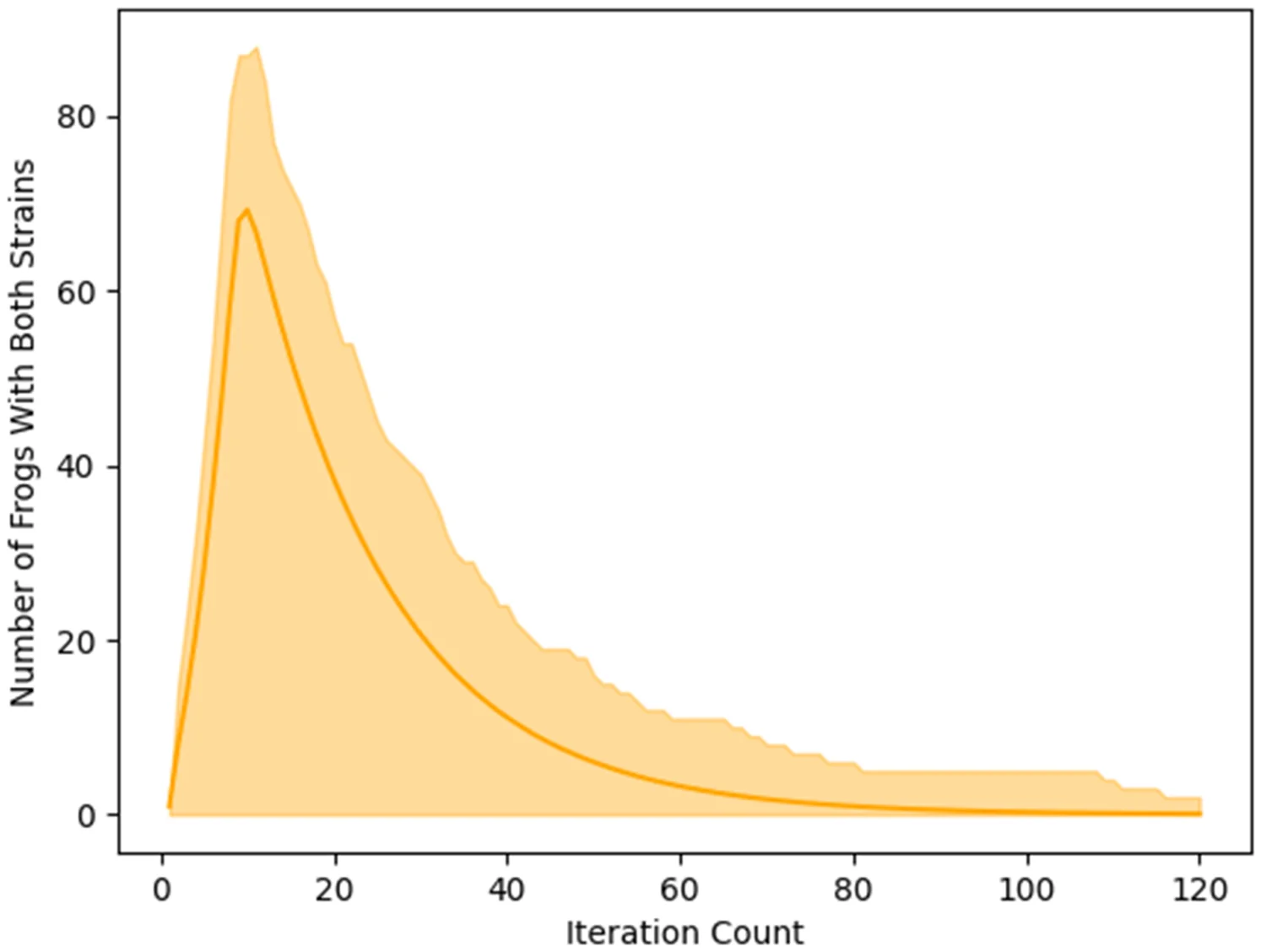
The darker orange curve is the mean value of the number of frogs in state ***C*** for each iteration; the shaded region goes from the minimum over all 10,000 ponds to the maximum.

We include the individual graphs for the number of frogs in each of the states ***U***, ***H***, and ***C*** over the course of the 120 iterations, with ranges showing the high and low values among the 10,000 ponds for clarity. Please see Figure 5, Figure 6 and Figure 7 below.

## 4. Regarding Death and Recruitment

Reason suggests that in the absence of the virus or other serious existential threats, a normal frog population would remain somewhat stable. This means that the mortality rate among frogs in state ***S*** is at the very least insignificant and does not play a major role in influencing the spread of the infection. We chose fifteen percent because that figure corresponds roughly to the 81% decline in population over 10 years observed by Teacher et al. [5]; when 15% die each year, we have 85% survive each year, and $0.85^{10}=0.197,$ so that 80.3% have died. (We rounded to the nearest 1% because of the underlying uncertainty in the original measurement of 81% fatality over 10 years.) We expected that the rate at which new frogs hatched and matured (i.e., were recruited into the population) should roughly compensate for natural death in the absence of the virus. However, it should be noted that natural mortality rates for common frogs have been suggested to be approximately 20% [28].

To make the model reflective of the situation post-initial emergence, we add a new parameter, ω, representing the mortality rate of infected frogs. The parameter ***ω*** may differ between syndromes, but until data are available, we make the assumption that the two syndromes are approximately equally lethal, and that frogs with both syndromes are not more likely to die than frogs with only one. We then adjusted this new variable accordingly to fit with the drastic population decline. With a mortality rate of 10%, we found that the mean time until all frogs were infected was 9.945; with 15%, as mentioned above, it was 10.02; and with 20%, it was 9.74. The effect of increasing the mortality rate, while holding the other parameters constant, is shown in Table 1. The effect of varying $\mu_{U}\;\mathrm{and}\;\mu_{H}$ can be seen in Table 2. It is important to note that this model is a short-term closed population outbreak model. Hence, we omit the effects of recruitment, natural mortality, migration, and detection rates.

In Table 2, below, we leave ω=0.15, but vary $\mu_{U}\;\mathrm{and}\;\mu_{H}$ by various values to simulate the effects of different contact rates. This means that, for instance, setting ψ=0.75 will produce values $\mu_{U}=0.75\cdot 0.65=0.4875$ and $\mu_{H}=0.75\cdot 0.75=0.6375$.

**Table 2:** Again, we ran the simulation with 10,000 ponds and 120 iterations. The values for ψ reflect the probabilities that the two frogs interact, and accordingly affect the probabilities of disease transmission. The rows for “All Infected,” “All Dead,” and “Pond Count” are as in Table 1.

ψ	0.75	0.80	0.85	0.90	0.95
$\mu_{U}$	0.4875	0.52	0.5525	0.585	0.6175
$\mu_{H}$	0.5625	0.60	0.6375	0.675	0.7125
All Infected	11.01	10.91	10.63	10.35	10.23
All Dead	38.53	38.29	37.99	37.85	37.79
Pond Count	9234	9395	9453	9472	9587

## 5. Notes on Contacts, Iterations, and Possible Expansions of the Model

Throughout this paper, we have used contact rates and iterations as generalized measures, as both have not been studied in depth in the UK at this time, and this is a weakness in our understanding of the model terms. The contact rates for common frogs that are affected by ranavirus would proceed in more realistic terms. Also, iterations used in the model should be considered as days or weeks, not months or years, since ranavirus infections can cause disease and mortality relatively quickly under laboratory conditions in adult frogs [24,25]. We can consider adjusting the probabilities of death if there is evidence that one syndrome is more lethal than the other, or whether a frog with both syndromes is more likely to die than a frog with only one. Possible expansions of the model could be from individuals to ponds; however, a distance measure would have to be added as ponds are rarely distributed evenly in the urban environment (Duffus personal observation). This would help understand how the spread of ranaviruses between sites occurred in the UK with potential applications in other regions. However, much more research is needed on contact rates and the length of time the virus takes to develop under natural conditions. Additionally, we require a greater understanding of how the different disease states are related in terms of their origins as a quasispecies, strain, or an isolate. In addition to this, Price et al. [29] suggest that climate change will alter ranavirus dynamics in the UK, and hence, temperature could also be incorporated into the model as a variable.

## 6. Brief Discussion and Conclusions

The cellular automata model of ranavirus spread is not totally consistent with results of the SI model presented by Duffus et al. [11]. Here, in the Hop ‘Til You Drop (HTYD) model, there are no survivors after approximately 38 iterations, whereas it took 65 years for the adult frog population to succumb to infection in the SI model. One key difference between the SI and HTYD models is that HTYD uses a slightly higher disease-induced mortality (15% per year) rather than the 8.1% disease-induced population decline that Duffus et al. [11] used. It is worth noting that a probability of death of about 10% led to the largest number of iterations before all frogs were dead, and that either higher or lower death rates appeared to reach universal death earlier. We hypothesize that, when the probability of death is low, a given frog has more time in which to infect others, but those other infected frogs will survive longer; when the probability of death is higher, the infected frogs die earlier, and this will slow the overall progress of the infection as dead frogs do not infect others. We also note that a death probability of 15% gave us the largest time at 10.2 iterations before all frogs were infected. Notice that the nature of the algorithm guarantees that some frogs must remain uninfected until the 8th iteration, because the infection can spread at only one row and column per iteration (and we started at column 8 and row 8). It seems likely that these values of the probability of death that led to the maximum number of iterations before universal death or universal infection are dependent upon the probabilities of transmission, and this might be worth exploring in further work.

Ultimately, the Hop ‘Til You Drop model demonstrates that incorporating spatial structure and probabilistic local interactions yields a vastly accelerated timeline for localized population collapse compared to traditional deterministic frameworks. By capturing the stochastic realities of individual contact within a finite grid, this cellular automaton approach highlights the extreme vulnerability of isolated garden pond populations to rapid extirpation. Although parameters such as contact rates and transmission probabilities remain open to refinement through empirical field studies, the model provides a flexible framework for future ecological predictive tools. Incorporating additional real-world variables, such as temperature-driven dynamics or inter-pond migration distances, will further elevate the model’s utility. As emerging infectious diseases continue to threaten global amphibian biodiversity, adaptive modeling strategies like the HTYD framework will be vital to understanding and mitigating the long-term impacts of ranavirosis.

## Figures and Tables

**Table 1:** We ran the simulation with 10,000 ponds and 120 iterations. The row labeled “All Infected” is the mean number of iterations before every frog had some form of the virus. The row labeled “All Dead” is the mean number of iterations among those ponds that had been entirely depopulated by the end of 120 iterations. The last row is the number of ponds out of 10,000 that had been entirely depopulated. The distribution is very skewed for higher values of ***ω***.

*ω*	0.075	0.100	0.125	0.150	0.175	0.200	0.225	0.250
All Infected	9.84	9.95	10.01	10.02	9.97	9.74	9.41	8.84
All Dead	71.12	54.41	44.39	37.64	37.79	29.39	26.85	24.76
Pond Count	9832	9904	9799	9626	9368	8950	8392	7670

## Data Availability

The original contributions presented in this study are included in the article and Python code in the Supplementary Material. Further inquiries can be directed to the corresponding author.

## Supplementary Materials

The following supporting information can be downloaded at: https://www.mdpi.com/article/10.3390/pathogens15080827/s1. File S1.
